# Simultaneous Quantification of Methotrexate and Its Metabolite 7-Hydroxy-Methotrexate in Human Plasma for Therapeutic Drug Monitoring

**DOI:** 10.1155/2019/1536532

**Published:** 2019-02-03

**Authors:** Xinxin Ren, Zhipeng Wang, Yunlei Yun, Guangyi Meng, Xialan Zhang, Huamin Ding, Ying Xu, Hansheng Bai, Jing Liu, Xia Li, Shouhong Gao, Lifeng Huang, Wansheng Chen

**Affiliations:** ^1^Department of Pharmacy, Changzheng Hospital, Second Military Medical University, Shanghai 200003, China; ^2^Center for Molecular Medicine, Xiangya Hospital, Key Laboratory of Molecular Radiation Oncology of Hunan Province, Central South University, Changsha 410008, China; ^3^Department of Pharmacy, The First People's Hospital of Yulin City, Yulin 537000, China; ^4^Department of Pharmacy, Suzhou Hospital of Traditional Chinese Medicine, Suzhou 215009, China; ^5^Department of Pharmacy, Shanghai Punan Hospital of Pudong New District, Shanghai 200125, China; ^6^Department of Pharmacy, The 97th Hospital of CPLA, Xuzhou 221004, China; ^7^Department of Pharmacy, The Second Hospital of Dalian Medical University, Dalian 116023, China; ^8^Department of Pharmacy, Yueyang Hospital of Integrated Traditional Chinese and Western Medicine, Shanghai University of Traditional Chinese Medicine, Shanghai 200437, China; ^9^Drug and Equipment Section, The 413th Hospital of CPLA, Zhoushan 316000, China

## Abstract

**Objective:**

To establish and validate a simple, sensitive, and rapid liquid chromatography tandem mass spectrometry (LC-MS/MS) method for the determination of methotrexate (MTX) and its major metabolite 7-hydroxy-methotrexate (7-OH-MTX) in human plasma.

**Method:**

The chromatographic separation was achieved on a Zorbax C_18_ column (3.5 *μ*m, 2.1 × 100 mm) using a gradient elution with methanol (phase B) and 0.2% formic acid aqueous solution (phase A). The flow rate was 0.3 mL/min with analytical time of 3.5 min. Mass spectrometry detection was performed in a triple-quadruple tandem mass spectrometer under positive ion mode with the following mass transitions: m/z 455.1/308.1 for MTX, 471.0/324.1 for 7-OH-MTX, and 458.2/311.1 for internal standard. The pretreatment procedure was optimized with dilution after one-step protein precipitation.

**Results:**

The calibration range of methotrexate and 7-OH-MTX was 5.0-10000.0 ng/mL. The intraday and interday precision and accuracy were less than 15% and within ±15% for both analytes. The recovery for MTX and 7-OH-MTX was more than 90% and the matrix effect ranged from 97.90% to 117.60%.

**Conclusion:**

The method was successfully developed and applied to the routine therapeutic drug monitoring of MTX and 7-OH-MTX in human plasma.

## 1. Introduction

It was estimated that there were 385,700 new cases and 199,700 deaths of non-Hodgkin lymphoma (NHL) occurred in 2012 worldwide [1]. The incidence of NHL increased in the majority of more developed countries up to 1990 and leveled off thereafter [2, 3]. High-dose methotrexate (MTX) plus calcium folinate (CF) is one of the classic therapeutic regimens for the treatment of NHL. MTX is a folate analogue that competitively inhibits dihydrofolate reductase and subsequently blocks the formation of tetrahydrofolate from dihydrofolate. Tetrahydrofolate is a one-carbon donor for the nucleic acid synthesis [4]. Also, MTX could suppress the polyamine formation and methylation of proteins and phospholipid [5–7]. The pivotal metabolite of MTX, 7-hydroxy-methotrexate (7-OH-MTX), was proven* in vivo* to decrease the intracellular concentration of MTX [8, 9]. The accumulated concentration of 7-OH-MTX might also influence the pharmacokinetics of MTX due to competitive transportation across cell membranes [10, 11], and the relationship between 7-OH-MTX and nephrotoxicity and hepatotoxicity had also been reported [12].

High-dose MTX (1-5 g/m^2^) could penetrate the blood-brain barrier, blood-eye barrier blood-testis barrier, and even the tumor cells to overcome the drug-resistance [13]. As a result, the high-dose MTX is prescribed preferably to the patients suffered from NHL, osteosarcoma, choriocarcinoma, and acute lymphoblastic leukemia in clinical [14]. As a nontargeting agent, high-dose MTX is restricted with a narrow therapeutic window, especially in the renal or liver impaired patients. Normal cells in rapid proliferative period could also been suppressed by the MTX. The side effects of MTX include nephrotoxicity, hepatotoxicity, myelosuppression, and so on [15]. The incidences of nephrotoxicity, myelosuppression, and blood toxicity were 20%, 80%, and 33%, respectively [16, 17]. An early report showed a 6% death incidence because of high-dose MTX [18]. Regarding precaution against the severe side effects after administration of high-dose MTX, the CF will be prescribed to alleviate the nucleic acids suppression sometimes, but an inopportune rescue may decrease the treatment efficacy or even enhance the side effects incidence. In clinical, new target of balance the treatment efficacy and the side effects has attracted the attentions of clinicians, who devote themselves to optimize the dose and administration time of CF to guarantee the efficacy and prevent the side effects. To achieve this goal, therapeutic drug monitoring for MTX had been carried out in clinical based on the immunoassay method, high performance liquid chromatography (HPLC) method, and HPLC tandem mass spectrometry method. For immunoassay method, the crosstalk between MTX and its metabolite 7-OH-MTX, high cost and narrow monitoring range have hindered its clinical application [19], and the endogenic compounds could also disturb the* in vivo* quantification of MTX [20]. The HPLC methods showed disadvantages of long analytical time and low sensitivity. Recent years have witnessed the emergence of LC-MS/MS methods for the monitoring of MTX exposure [21–30]. Some advantages, for example, short analytical time, high sensitivity, simple pretreatment procedure, and high universality for multiple drugs, had been individually shown by these reported LC-MS/MS methods, but a better clinical application could be achieved if these advantages were systematically integrated in one method. Here, we report a newly developed and validated UHPLC-MS/MS method for the simultaneous determination of MTX and 7-OH-MTX in human plasma and the application was completed on clinical samples from NHL patients.

## 2. Experimental

### 2.1. Chemicals and Reagents

**Figure 1:**
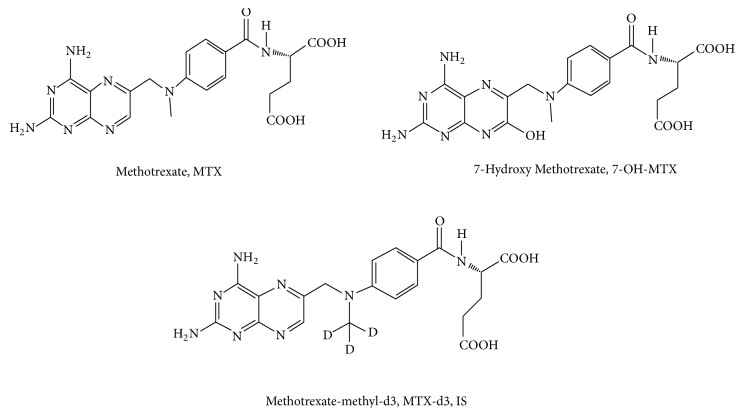
The chemical structures of MTX, 7-OH-MTX, and MTX-d_3_.

The standards including MTX, 7-OH-MTX, and MTX-d3 were purchased from Dalian Meilun Biotech Co., Ltd. (Dalian City, China. Purity >98% for MTX, 7-OH-MTX and MTX-d3. Lot No. MB1156-S, J0211A, J1213A). Chemical structures of MTX, 7-OH-MTX, and MTX-d3 are shown in Figure 1. HPLC-grade methanol and acetonitrile were both bought from Merck (Merck Company, Darmstadt, Germany). Formic acid was purchased from Tedia Company and of HPLC grade (Tedia Fairfield, OH, USA). Deionized water was prepared using a Milli-Q Reagent Water System in laboratory (Millipore, MA, USA). HPLC-grade ammonium hydroxide was obtained from Rowe Scientific Pty Ltd. (Perth, Australia). All other reagents were of analytical grade.

### 2.2. Instrumentation

All experiments were performed on an Agilent 6460 triple-quadrupole mass spectrometer equipped with an electrospray ionization source (Agilent Technology, MA, USA). The chromatographic separation was carried out on Agilent 1290 series ultra-high performance liquid chromatography, which contains a quaternary pump, an online degasser, an oven, and an autosampler. The instruments were operated with MassHunter software (Version 6.0.0, Agilent Technology, MA, USA).

### 2.3. Liquid Chromatography and Mass Spectrometry Conditions

**Figure 2:**
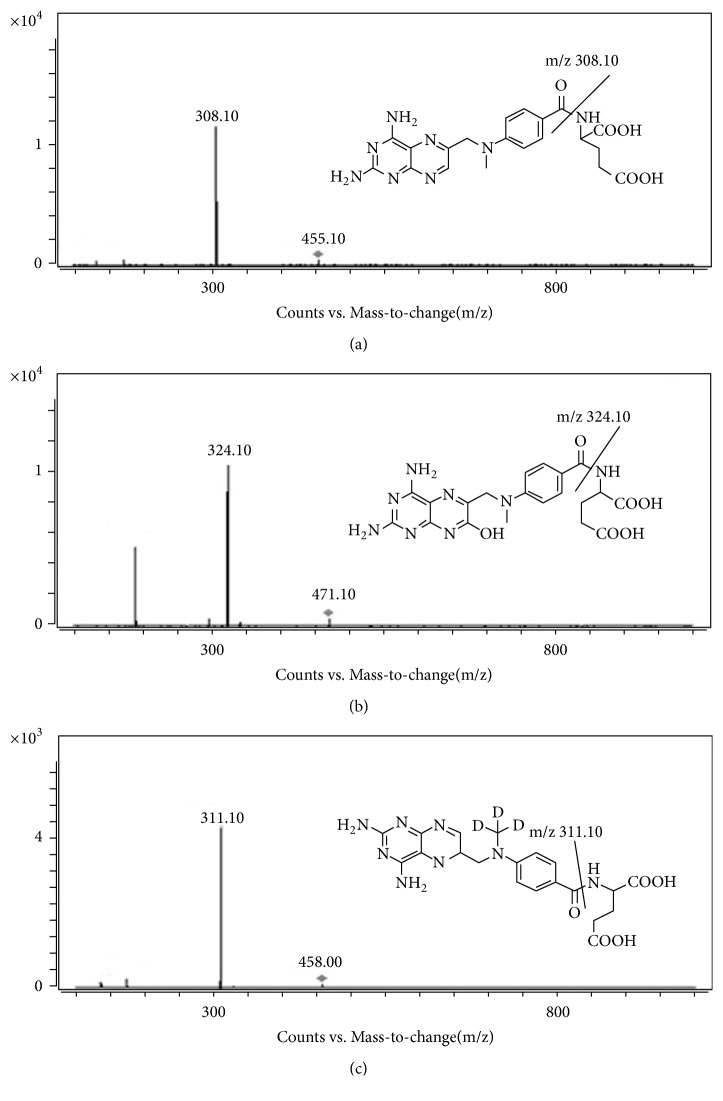
The product ion plots and fragmentor structures of MTX, 7-OH-MTX, and IS in positive mode. (a) MTX; (b) 7-OH-MTX; (c) IS.

Chromatographic separation was achieved on an Agilent ZORBAX C_18_ column (2.1 × 100 mm, 3.5 *μ*m) and temperature was maintained at 35°C. The mobile phase included solvent A (0.2% formic acid in water) and solvent B (methanol) and was delivered at 0.3 mL/min. The gradient elution started at 8% B and varied as follows: 0-1 min: 8%-30% B; 1-2 min: 30%-60% B; 2-3 min: 60%-70% B; 3-3.5 min: 70%-70% B. The column was equilibrated with initial phase for 3 min before first injection. The injection volume was 5 *μ*L and the total run time was 6.5 min. Mass spectrometry detection of analytes and IS was performed on Agilent 6460A, a triple-quadrupole mass spectrometer, operating in positive ionization mode. The quantification was performed using multiple reaction monitoring (MRM) mode with the following transitions: m/z 455.1/308.1 for MTX, m/z 471.0/324.1 for 7-OH-MTX, and m/z 458.2/311.1 for IS, respectively (Figure 2). The fragmentor voltages for MTX, 7-OH-MTX, and IS were optimized at 114 V, 110 V, and 114 V, respectively, and the collision energy was 13 eV, 8 eV, and 17 eV, respectively. The optimal mass spectrometry parameters were as follows: gas temperature 350°C; dry gas flow (N_2_) 10 L/min; nebulizer gas 50 psi; sheath gas temperature 300°C; sheath gas flow (N_2_) 12 L/min; capillary voltage 5000 V. For collision-induced dissociation, high-purity nitrogen was utilized as collision gas and delivered at a pressure of 0.2 MPa. Quadrupole 1 and quadrupole 3 were maintained at unit resolution. Dwell time was 200 ms for all analytes.

### 2.4. Preparation of Stock and Working Solutions

The stock solutions of MTX and IS were prepared in methanol (including 0.1% ammonium hydroxide) both at 1.0 mg/mL, and for 7-OH-MTX, it was prepared in methanol-ammonium hydroxide-water (7:2:1, v/v/v) solution to obtain a final concentration of 1.0 mg/mL. All stock solutions were stored at −80°C until analysis. The work solutions of analytes and the IS (500 ng/mL) were freshly prepared by further diluting the stock solutions with methanol-water (1:9, v/v).

### 2.5. Preparation of Calibration Standards and Quality Control Samples

Calibrations standards were prepared by adding appropriate volume of the work solutions into blank human plasma at concentrations of 5, 10, 100, 200, 500, 2000, 5000, and 10000 ng/mL for MTX and 7-OH-MTX. The quality control (QC) samples were separately prepared in the same way to obtain concentrations of 10 (LQC, low), 500 (MQC, medium), and 5000 (HQC, high) ng/mL for MTX and 7-OH-MTX. All samples were stored at −80°C.

### 2.6. Sample Pretreatment

To a 100 *μ*L aliquot of sample, 20 *μ*L IS solution (500 ng/mL in methanol-water solution, 1:9, v/v) and 300 *μ*L methanol were added. The mixture was vortex-mixed for 3 min and centrifuged at 13600 ×g for 5 min at room temperature. Then 100 *μ*L of the supernatant was transferred to 400 *μ*L 20% methanol aqueous solution. After vortex-mixing for 1 min, the mixture was centrifuged again with the same conditions. A 5 *μ*L aliquot of supernatant was injected into the LC-MS/MS system for analysis.

### 2.7. Method Validation

#### 2.7.1. Specificity

Specificity was evaluated by comparing the chromatograms from six different lots blank samples with the spiked samples, and the corresponding responses less than 20% of LLOQ and 5% of IS were considered reasonable.

#### 2.7.2. Recovery and Matrix Effect

The recovery was assessed by comparing the peak areas of the spiked samples with the spiked postextraction samples at the same concentrations (n=3), and the matrix effect was estimated by comparing the peak areas of spiked postextraction samples to the water-substituted samples at the same concentrations (n=3). Both recovery and matrix effect were evaluated at low, medium, and high concentrations of the analytes. MTX and 7-OH-MTX were assessed at 10, 500, and 5000 ng/mL, and IS was investigated at a single concentration of 500 ng/mL.

#### 2.7.3. Linearity and Lower Limit of Quantification (LLOQ)

The calibration curves of MTX and 7-OH-MTX ranged from 5 to 10000 ng/mL and nine calibration standards were prepared in this range for both analytes. The calibration curves were regressed by plotting the peak area ratio of analyte to IS versus corresponding concentrations using a 1/*x*^2^ weighted linear least-squares regression model (n=5). The LLOQ was defined as the lowest concentration point on the calibration curve which should meet the precision and accuracy criterions (n=5), and the deviation of back-calculation for the other concentration points should not go beyond ±15% except for LLOQ, which has a more extensive deviation to ±20%.

#### 2.7.4. Interday and Intraday Precision and Accuracy

The intraday and interday precision and accuracy were evaluated by analyzing five replicates of QC samples at three concentration levels in separated days (at least 2 days). Precision was expressed as the relative standard deviation (RSD%). The accuracy was determined as the deviation between the measured concentrations and the nominal concentrations. The RSD% for interday and intraday precision not more than 15% was rational. For intraday and interday accuracy, RE% (relative error) within ±15% was considered to be acceptable.

#### 2.7.5. Stability

The stability, which included freeze-thaw stability, short-term stability, long-term, and bench-top stability of MTX, 7-OH-MTX, and IS in plasma, was investigated. Freeze-thaw stability was assessed after QC samples freeze-thaw for three cycles. Bench-top stability was assessed by analyzing the QC samples prepared and placed on the bench for 6 h. Long-term stability was tested by analyzing the QC samples stored at –80°C for 30 days. Short-term stability was evaluated by analyzing the extracted QC samples kept in the autosampler at 4°C for 24 h. The concentrations of analytes were calculated using accompanying constructed calibration curve.

#### 2.7.6. Carry-Over

Carry-over of MTX and 7-OH-MTX was also tested by injecting the highest calibration standard sample prior to injecting a blank sample, and the responses of analytes in blank samples not more than 20% of the LLOQ and 5% of the IS were considered to be acceptable.

#### 2.7.7. Dilution Effect

The dilution effect was assessed by comparing the measured concentration to the nominal dilution concentration. Freshly prepared spiked samples with concentration beyond the highest calibration standard should be diluted by corresponding blank matrix into the linear range. Every dilution factor should be assessed at least for five times, and the RSD% and RE% should not more than 15% and within ±15%.

### 2.8. Clinical Application for MTX Exposure Monitoring

The experimental protocol was reviewed and approved by the Ethical Committee of Changzheng Hospital (Shanghai, China) and carried out in Changzheng hospital. Informed consent was signed by all the participants. Finally, 11 malignant lymphoma patients (male 5; female 6) in total were recruited and the samples were collected at 44, 68, and 92 h for every patient after administration of MTX using intravenous drip. The average age was 53.45 ± 9.75 (Mean ± SD) years. The dosage of MTX for the 11 patients was 0.77~3.50 g/m^2^ based on their clinical diagnosis. The samples were shaken slightly and then centrifuged at 3000 ×g for 10 min. The supernatant (plasma) was harvested and stored at −80°C until analysis.

## 3. Results and Discussions

### 3.1. LC-MS/MS Condition Optimization

LC-MS/MS method is based on the chromatography separation and mass spectrometry detection. An optimized chromatography separation and mass spectrometry detection could facilitate the drug quantification and analysis process. The first way to achieve the goal is to find a better mobile phase and the buffer substance, and the buffer substances could adjust the mobile phases pH and enhance the ionization efficiency to obtain a better peak symmetry and higher responses [31]. For the condition optimization, different buffer substances, for example, formic acid (0.05%, 0.1%, 0.2%, and 0.5%; V/V), acetic acid (0.1%; V/V), ammonium acetate (5 mmol/L, 10 mmol/L), were added for the tailing suppression and response enhancement. In addition, the organic reagents including methanol and acetonitrile and their mixture in different proportions were tested for the elution capacity and peak symmetry. As a result, 0.2% formic acid in water phase and methanol was proven to be the optimal mobile phases for the MTX and 7-OH-MTX separation. Also, different columns containing Poroshell 120 SB-C_18_ (2.1 mm × 75 mm, 2.7 *μ*m), ZORBAX C_18_ (2.1 × 100 mm, 3.5 *μ*m), Inertsil ODS-3 (2.1 mm × 50 mm, 5 *μ*m), XBridge BEH C_18_ (3.5 *μ*m, 2.1 × 100 mm), and XSelect CSH C_18_ (3.5 *μ*m, 2.1 × 100 mm) were tested for the retention and reproductivity of MTX and 7-OH-MTX, and the ZORBAX C_18_ (2.1 × 100 mm, 3.5 *μ*m) showed an optimal result. Finally, the ion source conditions were optimized to gain a better MS response as mentioned above.

### 3.2. Sample Preparation

For LC-MS/MS development, the extraction and purification of analytes were most challenging and it was reported that approximately 61% of time was assigned to the extraction and purification process in a LC-MS/MS development [32]. The one-step protein precipitation used in this method was a simple, efficient, and economical way which has been widely reported by some researchers [4, 30, 33], while solid phase extraction and liquid-liquid extraction were more time-consuming and wasteful. To further improve the detection sensitivity, we diluted the supernatant after the protein precipitation with different proportion of water-methanol mixture. In this step, 8% (initial phases), 20%, 50%, and 70% methanol aqueous solution were added to the supernatant in different proportions (1:1, 1:2, 1:3, 1:4, 1:5; V/V). The results showed that the highest sensitivity could be obtained after a 5 times' dilution with the 20% methanol aqueous solution to the supernatant and the sensitivity was enhanced to 5 ng/mL for MTX and 7-OH-MTX.

### 3.3. Method Validation

#### 3.3.1. Specificity

**Figure 3:**
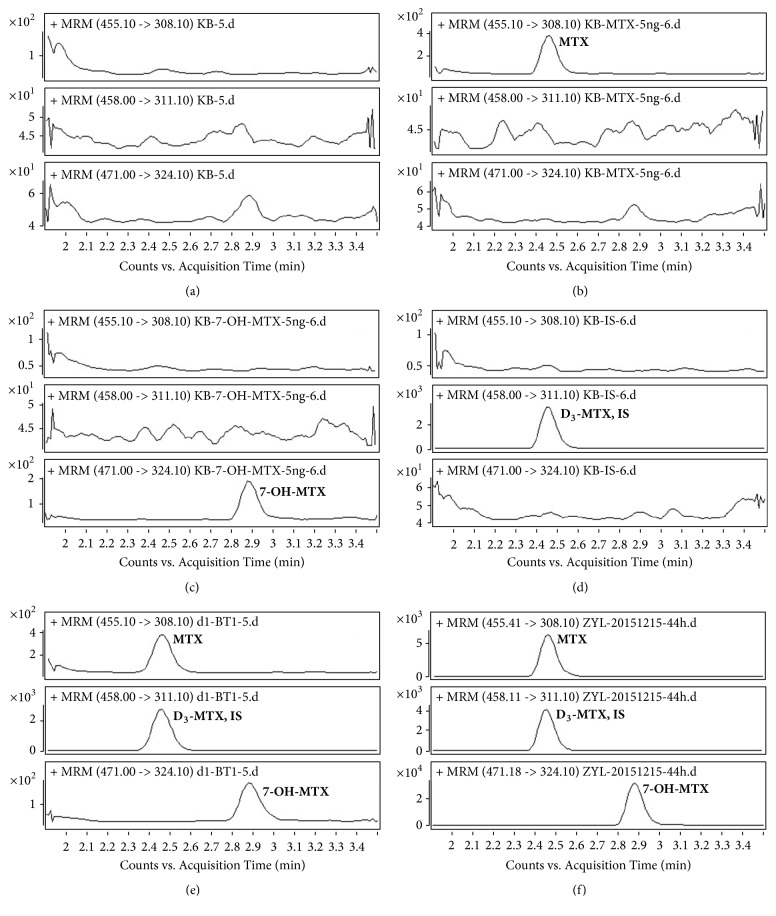
The chromatograms of MTX, 7-OH-MTX, and IS under MRM mode: (a) blank sample; (b) blank plasma spiked with MTX (5 ng/mL); (c) blank plasma spiked with 7-OH-MTX (5 ng/mL); (d) blank plasma spiked with IS; (e) lower limit of quantification sample; (f) real sample collected at 44 h after the administration of MTX from one patient.

Figure 3 showed the MRM chromatograms of blank sample, analytes spiked sample at LLOQ concentration, IS spiked sample, and real sample. The retention times of MTX, 7-OH-MTX, and IS ranged from 2.46 to 2.88 min. No significant interferences were observed in human plasma at the retention times of MTX, 7-OH-MTX, and IS.

#### 3.3.2. Recovery and Matrix Effect

The recovery and matrix effect of MTX, 7-OH-MTX, and IS were presented in Table 1. Recovery of MTX and 7-OH-MTX at different concentration levels were 92.47%-97.87% and 91.45%-97.61%, respectively, and all the RSD% were less than 15%. The results indicated that the extraction process was stable and efficient. The mean matrix effects were 116.07%-117.60% for MTX and 97.90%-102.96% for 7-OH-MTX at the three concentration levels, respectively, which showed that the ionization suppression or enhancement from the human plasma matrix was stable and acceptable in the current method.

#### 3.3.3. Linearity and LLOQ

Linearity and LLOQ are summarized in Table 2. The regression equation was constructed using* y = ax+b* formula, where *y* represents the peak area ratio of analytes to IS and *x* is the nominal concentration of analytes. The correlation coefficients (*r*) were > 0.99 for MTX and 7-OH-MTX. The results showed a good linearity in the concentration range. The lowest concentrations on the calibration curve were the LLOQ for MTX and 7-OH-MTX, respectively. The LLOQ was sufficient for the therapeutic drug monitoring of MTX and 7-OH-MTX in clinical practice.

#### 3.3.4. Interday and Intraday Precision and Accuracy

The intraday and interday precision and accuracy of analytes at three concentration levels were summarized in Table 3. The intraday precisions were 0.89%-2.47% and 1.90%-6.86% for MTX and 7-OH-MTX, respectively. The interday precisions were 1.84%-3.41% and 3.19%-6.40% for MTX and 7-OH-MTX, respectively. The interday and intraday accuracy of MTX and 7-OH-MTX were within ±15%. The results indicated that the method had an acceptable reproducibility (Table 3).

#### 3.3.5. Stability

The analytes had acceptable stabilities after three freeze-thaw cycles, being in room temperature for 6 h, being stored at −80°C for 30 days, and being in autosampler at 4°C for 24 h. The results of all QC samples deviated within ±15%, as shown in Table 4.

#### 3.3.6. Carry-Over

The results displayed an obviously carry-over for MTX and 7-OH-MTX as the responses in the blank sample injected after the highest sample were more than 20% of the LLOQ responses. To solve this problem, a blank sample was added following a high concentration sample to elute the analytes residuals, and the carry-over of MTX and 7-OH-MTX was less than 20% of the LLOQ after the elution process.

#### 3.3.7. Dilution Effect

For dilution effect assessment, a 20000 ng/mL spiked sample was freshly prepared and diluted for 5 times to 4000 ng/mL with blank plasma. After being assessed for 5 times, the RE% and RSD% of the dilution factor were less than 15% and within ±15%, which conformed to the criterions.

### 3.4. Application of the Method

**Figure 4:**
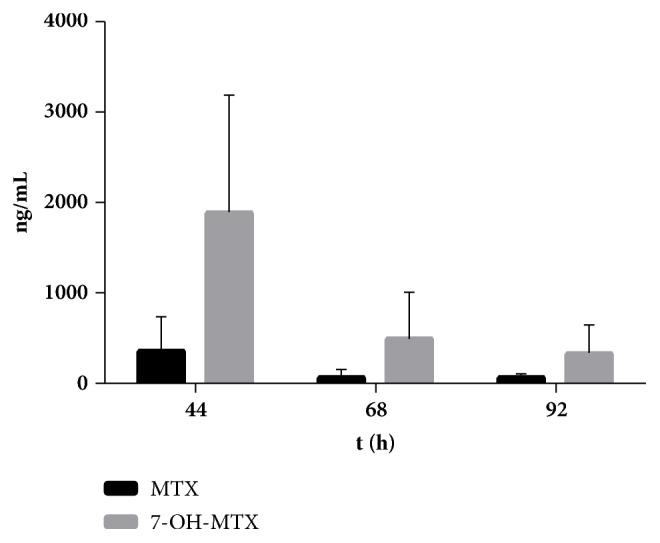
Determined concentrations of MTX and 7-OH-MTX (Mean ± SD).

Finally, 49 samples were collected and quantitatively analyzed with this newly developed method. After administration, 22 samples were collected at 44 h; 21 sample were gathered at 68 h; and the other samples were harvested at 92 h. Research had suggested a safe concentration window for the MTX exposure [34] and our results showed that 31.82% of the patients suffered from excessive exposure at 44 h to MTX, and at 68 h, the patients with a higher MTX exposure than threshold account for 38.09%. Besides, the* in vivo* exposure of 7-OH-MTX was much higher than MTX at all three sampling points, which was consistent with previously reported results [26]. Furthermore, An overhigh 7-OH-MTX concentration was believed to associate with the hepatotoxicity and nephrotoxicity after MTX therapy [35, 36]. Our results also displayed huge interpatient variations of MTX and 7-OH-MTX, which suggested that a timely and accurately therapeutic drug monitoring and rescue of MTX and 7-OH-MTX is necessary in clinical practice (Figure 4).

## 4. Conclusion

A sensitive, simple, and efficient UHPLC-MS/MS method has been successfully developed and validated for simultaneous determination of MTX and 7-OH-MTX in human plasma. The lower limit of quantification was 5 ng/mL for MTX and 7-OH-MTX. The analytical time was 3.5 min and the pretreatment procedure was developed based on a simple protein precipitation, which paved a way for high-throughput sample analysis. This method was applied on clinical samples from malignant lymphoma patients and the results were beneficial to avoid side effects after high-dose MTX therapy.

## Figures and Tables

**Table 1:** Recovery and matrix effect for the analytes in human plasma (n=3).

Analyte	Nominal concentration (ng/mL)	Recovery		Matrix Effect	
		Mean ± SD	RSD	Mean ± SD	RSD
		(%)	(%)	(%)	(%)
MTX	10	92.76 ± 2.50	2.70	116.38 ± 1.33	1.15
	500	97.87 ± 4.81	4.92	117.60 ± 7.07	6.01
	5000	92.47 ± 8.18	8.85	116.07 ± 1.26	1.08
7-OH-MTX	10	97.61 ± 4.69	4.80	102.96 ± 9.08	8.82
	500	95.17 ± 3.51	3.69	97.90 ± 7.28	7.42
	5000	91.45 ± 4.27	4.67	99.57 ± 4.94	4.96
IS	500	100.50 ± 6.16	6.13	116.24 ± 3.06	2.63

**Table 2:** Linearity regression parameters for MTX and 7-OH-MTX (n=5).

Analyte	Linearity Range (ng/mL)	Calibration curve	*r*	LLOQ (ng/mL)	Measured Concentration (ng/mL)	Accuracy (RE*∗*/%)
MTX	5~10000	*y*=11.4356*x*-0.0158	0.9977	5	5.23 ± 0.21	4.57
7-OH-MTX	5~10000	*y*=3.9966*x*+0.0028	0.9978	5	5.12 ± 0.56	2.35

*∗*RE is expressed as [(mean measured concentration)/(nominal concentration)-1]×100%

**Table 3:** Intraday and interday precision and accuracy of MTX and 7-OH-MTX in human plasma (n=5).

Analyte	Nominal concentration (ng/mL)	Intra-day(n = 5)			Inter-day(n = 15)		
		Measured concentration (ng/mL)	Precision (RSD%)	Accuracy (RE*∗*%)	Measured concentration (ng/mL)	Precision (RSD%)	Accuracy (RE%)
MTX	10	9.48 ± 0.23	2.47	-5.22	9.30 ± 0.32	3.41	-7.05
	500	514.84 ± 4.56	0.89	2.97	515.67 ± 9.49	1.84	3.13
	5000	5265.35 ± 92.88	1.76	5.31	5305.42 ± 118.95	2.24	6.11
7-OH-MTX	10	9.69 ± 0.66	6.86	-3.15	9.70 ± 0.62	6.40	-3.04
	500	456.43 ± 8.69	1.90	-8.71	469.16 ± 14.98	3.19	-6.17
	5000	4772.84 ± 267.84	5.61	-4.54	4772.93 ± 189.12	3.96	-4.54

**Table 4:** Stability of MTX and 7-OH-MTX in different conditions (n=5).

Analyte	Nominal concentration (ng/mL)	three freeze-thaw cycles (RE/%)	Short-term (RE/%)	Long-term (RE/%)	Bench-top (RE/%)
MTX	10	-5.60	-5.99	-2.27	-0.03
	500	4.02	1.56	-7.29	5.10
	5000	4.58	4.99	6.83	8.22
7-OH-MTX	10	-0.91	-5.96	-9.10	7.22
	500	-8.04	-8.66	-12.91	-0.77
	5000	-7.02	-5.80	-5.26	2.41

## Data Availability

The data used to support the findings of this study are included within the article.
